# Molecular Characterization and Pathogenicity of the Novel Recombinant Muscovy Duck Parvovirus Isolated from Geese

**DOI:** 10.3390/ani11113211

**Published:** 2021-11-10

**Authors:** Kuang-Po Li, Yu-Chen Hsu, Chih-An Lin, Poa-Chun Chang, Jui-Hung Shien, Hsien-Yueh Liu, Hua Yen, Shan-Chia Ou

**Affiliations:** 1 R&D Section, Wonder Biotek Co., Ltd., Pintung 908, Taiwan; vmcample@yahoo.com.tw; 2 Graduate Institute of Microbiology and Public Health, National Chung Hsing University, Taichung 402, Taiwan; tsula17@gmail.com (Y.-C.H.); yoshimep@gmail.com (C.-A.L.); pcchang@mail.nchu.edu.tw (P.-C.C.); 3 Department of Veterinary Medicine, National Chung Hsing University, Taichung 402, Taiwan; jhshien@dragon.nchu.edu.tw; 4 Bachelor Degree Program in Animal Healthcare, Hung Kuang University, Taichung 433, Taiwan; lhy_vet@sunrise.hk.edu.tw

**Keywords:** goose parvovirus (GPV), Muscovy duck parvovirus (MDPV), recombination

## Abstract

**Simple Summary:**

Waterfowl parvoviruses are important pathogens that cause severe disease in young waterfowl. Waterfowl parvoviruses can be divided into goose parvovirus (GPV)- and Muscovy duck parvovirus (MDPV)-related groups. New variant strains can be generated from genomic recombination between different waterfowl parvoviruses and result in new epidemics. Recently, a novel recombinant MDPV (rMDPV) derived from recombination between GPVs and MDPV was reported. This virus caused high morbidity and mortality rates in ducklings and was circulating in waterfowl in mainland China. In this study, a novel rMDPV was isolated in Taiwan from a goose flock that experienced a high mortality. The complete genome of this goose-origin rMDPV was sequenced. Phylogenetic and recombination analyses were performed to elucidate its molecular characteristics. The virulence of this rMDPV was evaluated using experimental infection goose embryos and goslings. This study was the first report showing the pathogenicity of rMDPV in geese.

**Abstract:**

Goose parvovirus (GPV) and Muscovy duck parvovirus (MDPV) are the main agents associated with waterfowl parvovirus infections that caused great economic losses in the waterfowl industry. In 2020, a recombinant waterfowl parvovirus, 20-0910G, was isolated in a goose flock in Taiwan that experienced high morbidity and mortality. The whole genome of 20-0910G was sequenced to investigate the genomic characteristics of this isolate. Recombination analysis revealed that, like Chinese rMDPVs, 20-0910G had a classical MDPV genomic backbone and underwent two recombination events with classical GPVs at the P9 promoter and partial VP3 gene regions. Phylogenetic analysis of the genomic sequence found that this goose-origin parvovirus was highly similar to the circulating recombinant MDPVs (rMDPVs) isolated from duck flocks in China. The results of experimental challenge tests showed that 20-0910G caused 100% mortality in goose embryos and in 1-day-old goslings by 11 and 12 days post-inoculation, respectively. Taken together, the results indicated that this goose-origin rMDPV was closely related to the duck-origin rMDPVs and was highly pathogenic to young geese.

## 1. Introduction

Waterfowl parvoviruses are highly contagious lethal pathogens for goslings and ducklings. Clinical infection can result in significant economic losses in countries with intensive waterfowl industries. Waterfowl parvoviruses can be divided into goose parvovirus (GPV)-related groups and Muscovy duck parvovirus (MDPV)-related groups, based on genetic characteristics, neutralization test results, and host ranges [1,2,3,4]. GPV, the agent of Derzsy’s disease, causes the disease in young geese and Muscovy ducks. In contrast, MDPV induces clinical signs so far found only in Muscovy ducks [5,6].

Both GPV and MDPV belong to *Anseriform dependoparvovirus 1* species, the *Dependoparvovirus* genus, and the Parvoviridae family [7]. Waterfowl parvoviruses contain a linear, single-stranded DNA genome approximately 5.1 kb in length. The protein-encoding regions, which are flanked by identical inverted terminal repeats (ITRs) at both ends, have two open reading frames (ORFs). The left ORF encodes the non-structural (NS) protein with viral replication functions. The right ORF encodes three structural capsid proteins, VP1, VP2, and VP3, which are derived from the same gene, and the coding regions of the VP2 and VP3 are included within the C-terminus of VP1 [2,3]. Phylogenetic analyses of VP genes revealed that waterfowl parvoviruses can be classified by geographical origin and viral pathogenicity. The nucleotide differences of VP1 between GPVs and MDPVs are about 20–24%; within the GPV and MDPV groups, nucleotide difference in VP1 are only about 0.1–7.0% and 0.1–1.9%, respectively [8,9,10,11]. 

Variant parvoviruses can be generated via recombination during virus evolution. Some variant canine parvoviruses (CPV) are derived from recombination between vaccine and field strains [12]. Canine parvovirus can recombine with feline panleukopenia virus or other feline parvovirus-like viruses to generate new types of variant strains [13]. Duck-origin novel goose parvoviruses (NGPVs) are circulating in duck farms that induce short beak and dwarfism syndrome (SBDS) in Cherry Valley ducks and mule ducks with high morbidity and low mortality in mainland China, Poland, and Egypt. These NGPVs could be recombinants derived from classical GPVs [14,15,16,17]. A new recombinant MDPV (rMDPV) that is generated from classical MDPV and exchanged a partial VP3 gene and the P9 promoter regions with classic GPVs was isolated in ducks from several provinces in mainland China. This rMDPV threatens Muscovy ducklings less than 3-week-old with high mortality and induces embolism in the intestinal tracts of infected ducklings, as GPV does in goslings. Serological tests indicated that this rMDPV has a closer antigen relationship with classical GPVs [18,19,20].

In Taiwan, two major outbreaks of waterfowl parvoviruses occurred in 1982 and 1989/1990. The 1982 outbreak was mainly caused by GPV and affected geese and Muscovy ducks. The 1989/1990 outbreak was mainly caused by MDPV, which affected many breeds of duck but geese were spared [4,9,21]. Since these outbreaks, a live attenuated vaccine has been used in goose breeders to protect goslings in the field from GPV via maternal antibodies. Therefore, although GPV is endemic, the annual mortality rate in affected farms is seldom above 5%. In 2020, a new type of waterfowl parvovirus with a mortality of 30% was detected from a 15-day-old goose flock in southern Taiwan. The objectives of this study were to isolate this goose-origin parvovirus and investigate its molecular characteristics and virulence in goslings.

## 2. Materials and Methods

### 2.1. Sample Collection, Detection, and Virus Isolation

Liver, spleen, and kidney samples that could contain large amounts of the virus were collected from 15-day-old White Roman goslings in a flock in Yunlin county in 2020. The breeders of this flock were vaccinated with an attenuated live GPV vaccine via intramuscular route before they entered the laying season. Infected goslings had clinical signs of diarrhea, growth retardation, and dyskinesia. The postmortem lesions of the infected birds included pale myocardium, congested liver, ascites, and necrotic enteritis. The morbidity and mortality rates were approximately 50% and 30%, respectively. The collected organs were homogenized in 10-fold volumes (based on weight) of PBS with antibiotics and centrifuged at 5000× *g* for 10 min. The supernatant was collected for virus isolation and nucleic acid extraction.

For virus isolation, the organ samples were inoculated into 12-day-old parvovirus-free embryonated Muscovy duck eggs via allantoic cavity. Allantoic fluid was harvested at 5–6 days post-inoculation, and the virus isolate was serial passaged five times in Muscovy duck embryos. 

Total nucleic acid from collected samples or virus isolates was extracted using a commercially available QIAmp^®^ DNA Mini Kit (Qiagen, Germany) according to the manufacturer’s instruction. Purified DNA was subjected to PCR assay for waterfowl parvovirus verification, as previously described [4].

### 2.2. Genome Cloning and Sequencing

To obtain the full-length genomic sequence, the genome was cloned into a pGEM-T Easy vector (Promega, Madison, WI, USA) using a TA cloning kit, as previously described by Yen et al. (2015) [22]. Briefly, purified DNA was annealed to the double-stranded form via heating at 95 °C for 3 min and 55 °C for 30 min. The 3′-A overhangs were added to the annealed DNA using Taq DNA polymerase. Five microliters of viral DNA was mixed with 5 μL 2× ligation buffer, 1 μL of pGEM-T vector (50 ng), and 1 μL T4 DNA ligase. The ligation mixture was incubated at 37 °C for 1 h and the ligated vectors were transformed into the *Escherichia coli* SURE strain (Stratagene Corporation, La Jolla, CA, USA). Recombinant plasmids from the transformants were purified using a QIAGEN^®^ Plasmid Mini Kit (Qiagen, Germany), according to the manufacturer’s instructions. Then, three randomly selected recombinant plasmids were submitted to Mission Biotech Inc. for sequencing using the primer sets, as previously described [19].

### 2.3. Sequence Analysis

Sequencing results were assembled using Lasergene v7.0 software (DNASTAR^®^, Madison, WI, USA). The sequences were aligned by the CLUSTAL W software of the MegAlign^TM^ program. Phylogenetic analysis of the sequences was performed with the maximum likelihood methods using the Kimura 2-parameters model and 1000 bootstrap replicates by MEGA version X software [23]. Potential recombination sites were identified using the Recombination Detection Program 4 (RDP 4) and default settings [24]. In this program, RDP, GENECONV, BootScan, MaxChi, Chimaera, SiScan, PHYLPRO, LARD, and 3Seq methods were provided to detect the recombination events and identify breakpoints of the recombinant sequences. A recombination event was accepted only if detected by at least four of these methods with a *p*-value ≤0.05. In addition, SimPlot version 3.5.1 was also used to further confirm the recombination results [25].

### 2.4. Determination of Mean Embryo Lethal Dose (ELD_50_) and Mean Embryo Infection Dose (EID_50_)

The virus was serial 10-fold diluted in PBS from 10^−1^ to 10^−7^. Two hundred microliters of each diluted virus was injected into 12-day-old parvovirus-free embryonated Muscovy duck eggs via allantoic cavity. Each dilution was used to infect five eggs. The eggs were incubated at 37 °C for 7 days. The embryos were examined for death or signs of hemorrhage and stunted growth. The results of embryo death or infection were used to calculate the ELD_50_ or EID_50_ value using the Reed and Muench method [26].

### 2.5. Experimental Infection and Virulence Assay

The viral virulence was evaluated in parvovirus-free White Roman goose embryos and goslings. All animal experiments were approved by the Institutional Animal Care and Use Committee of National Chung Hsing University (IACUC No.109-102) and were performed based on the ethical rules and laws of the University. Ten 12-day-old goose embryos were inoculated with 10^5^ EID_50_ of virus via the allantoic cavity. The eggs were incubated at 37 °C for 14 days and were candled daily. Survival rate was calculated and recorded. Twenty 1-day-old goslings were divided into two groups. In the first group, the birds were injected with 0.2 mL of virus via intramuscular route at a titer of 10^5^ ELD_50_. The birds in the second group were injected with 0.2 mL of PBS via the same route. Feed and water were provided ad libitum. Post-injection survival rates of experimental animals were recorded daily.

## 3. Results

### 3.1. Virus Isolation

The collected organ samples were inoculated into parvovirus-free Muscovy duck embryos for five serial passages. Waterfowl parvovirus was detected in the organs and in the harvested allantoic fluid using PCR assays. The samples were negative for other waterfowl pathogens, such as avian influenza virus, goose hemorrhagic polyomavirus, Tembusu virus, and waterfowl circovirus. This goose-origin parvovirus was designed as 20-0910G. The genomic sequence of 20-0910G was deposited in GenBank with an accession number of OK392126.

### 3.2. Nucleotide Sequence, Recombination, and Phylogenetic Analyses

The complete genome sequence of 20-0910G contained 5071 nucleotides in length. The right ORF, encoding VPs, consisted of 2199 nucleotides in length. The left ORF, encoding the NS protein, consisted of 1884 nucleotides in length. The ITRs at both ends of the viral genome consisted of 424 nucleotides. Compared with previously published waterfowl parvovirus sequences, the 20-0910G isolate had 99.7% sequence identity to the rMDPV (JH10 strain) isolated in mainland China [19].

**Figure 1:**
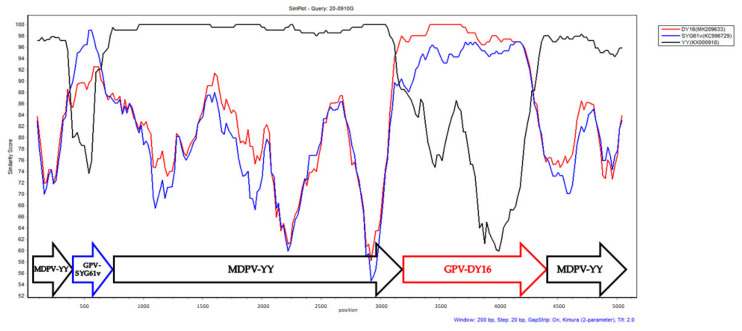
The Simplot analysis of the complete genomic sequences of GPV and MDPV. The 20-0910G isolate was used as the query. The YY, SYG61v, and DY16 strains were the potential parental strains. Two regions, at nucleotide positions 423–615 and 3121–4251, were found to contain the recombination breakpoints. The pairwise distance with a window size of 200 bp and step size of 20 bp were used for the analysis. The potential recombination breakpoints are located at the junction of forward arrows.

Recombination analysis was performed using RDP4 and SimPlot. Similar to rMDPVs isolated from mainland China, 20-0910G had a classical MDPV genomic backbone and underwent two recombination events with classical GPVs at the P9 promoter (nucleotide positions 423–615) and partial VP3 gene region (nucleotide positions 3121–4251) (Figure 1).

**Figure 2:**
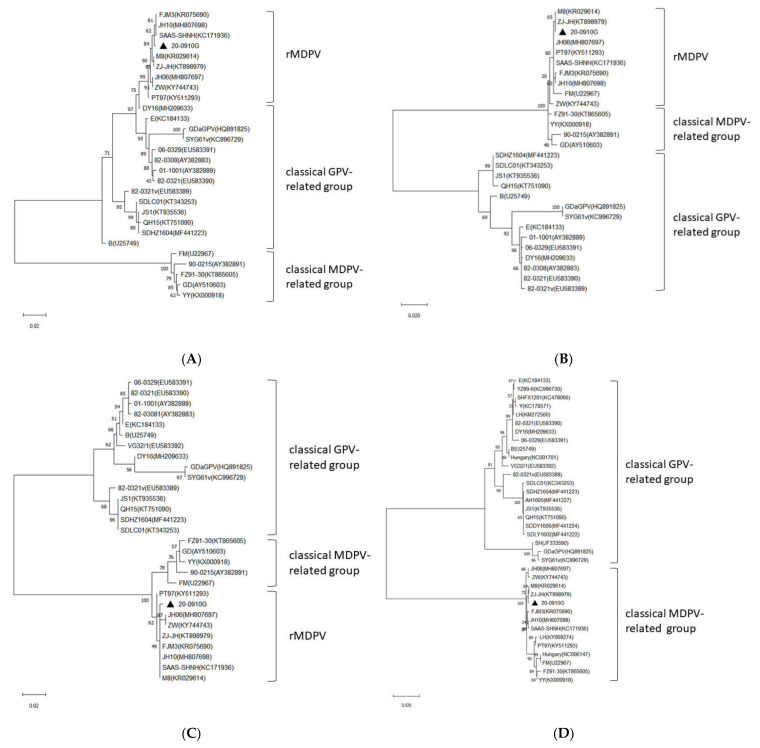
Phylogenetic analyses based on the nucleotide sequences from (**A**) the central 1.1-kb segment of VP1 (nucleotide positions 3121–4251); (**B**) the N-terminal 700-bp segment of VP1 (nucleotide positions 2419–3118); (**C**) the C-terminal 400-bp segment of VP1 (nucleotide positions 4218–4617); (**D**) the NS gene. All analyses were performed with the maximum-likelihood method. Relative bootstrap values were indicated at the nodes by 1000 replicates. Sequence determined in the present study is marked with a triangle.

On the basis of the result of recombination analyses, the VP1 gene from 20-0910G was split into three segments for phylogenetic analyses: the central 1.1-kb segment (nucleotide positions 3121–4251), the N-terminal 700-bp segment (nucleotide positions 2419–3118), and the C-terminal 400-bp segment (nucleotide positions 4218–4617). The phylogenetic tree based on the central 1.1-kb segment showed that 20-0910G clustered with rMDPVs and fell into the classical GPV group (Figure 2A). In contrast, phylogenetic trees based on the N-terminal 700-bp and C-terminal 400-bp segments revealed that 20-0910G clustered with rMDPVs and fell into the classical MDPV group (Figure 2B,C). These results confirmed that 20-0910G, like other rMDPVs, was originating from recombination between GPVs and MDPV. The VP1 nucleotide differences between 20-0910G and other rMDPV strains were 0.2–0.7%. The 20-0910G isolate had 9.9–11.8%, 10.7–11.3%, and 8.5–11.6% nucleotide differences from the classical GPV, MDPV, and NGPV groups, respectively.

Phylogenetic analysis of the NS gene revealed the presence of two main groups. The first group was GPV-related group which contained GPVs and NGPVs. The second was the MDPV-related group which contained MDPVs and rMDPVs. The 20-0910G isolate fell into the rMDPV group and showed 0.4–0.8% sequence distance from other rMDPV strains. The nucleotide distances between the NS gene of 20-0910G and the GPV, MDPV, and NGPV groups were 16.7–18.4%, 1–1.3%, and 17.7–18.4%, respectively (Figure 2D).

### 3.3. Virulence Assay of 20-0910G

**Figure 3:**
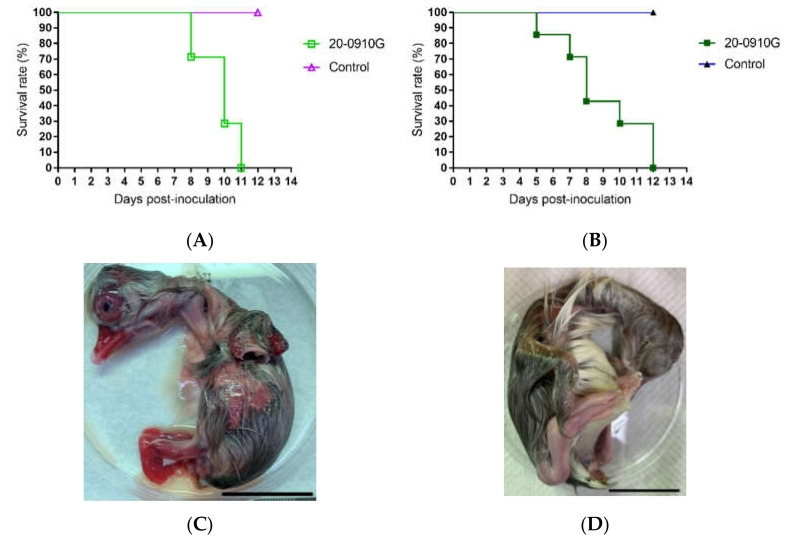
Pathogenicity of 20-0910G in goose embryos and goslings. The embryos were infected with 10^5^EID_50_ and 1-day-old goslings were infected with 10^5^ELD_50_ of 20-0910G. (**A**) Survival rate of goose embryos; (**B**) survival rate of goslings. All embryos and goslings died within 11 and 12 days post-infection, respectively. (**C**) 20-0910G infected embryos showed stunting and subcutaneous hemorrhage at 8 dpi; (**D**) a 20-day-old goose embryo used as control. The scale bar is 30 mm.

Experimental challenge tests were performed to evaluate the virulence of the 20-0910G isolate in White Roman goose embryos and goslings. All 12-day-old embryos that were infected with 10^5^ EID_50_ virus died within 11 days post inoculation (dpi); the mean death time (MDT) value was 9.7 days. All challenged embryos showed stunting and body hemorrhage. Similarly, the 10^5^ ELD_50_ infected 1-day-old White Roman goslings died within 12 days post-infection; and the MDT value was 8.86 days (Figure 3). All infected goslings showed the clinical signs of watery diarrhea, dyskinesia, growth retardation, and feather disorder. Taken together, these results indicated that the 20-0910G isolate was highly virulent for goslings.

## 4. Discussion

Our clinical observations found that atrophic bills with protruding tongues and growth retardation were common in survival ducklings after waterfowl parvovirus infections [21]. These clinical symptoms are very similar to those found in ducks infected with NGPVs. However, the NGPVs induced high morbidity but low mortality rates in young waterfowls [27]. GPV-infected goslings showed growth retardation and feather disorder with high mortality. In this study, we isolated a rMDPV from a young goose flock and showed that this virus caused a high mortality rate in geese. To the best of our knowledge, this is the first report showing the pathogenicity of rMDPV in geese.

The complete genome sequence of 20-0910G had 99.7% sequence similarity to the JH10 strain, an rMDPV isolated in China. Phylogenetic analyses found that the structural and non-structural gene sequences of 20-0910G were clustered within the rMDPV group. The rMDPV was first isolated in Chinese duck farms in the 1990s; the recombination occurred in the VP3 region. After circulating in the field about a decade, this rMDPV underwent another recombination event in the P9 promotor region [19,28,29,30]. These recombinant MDPVs spread quickly in waterfowl and formed a new clade of waterfowl parvoviruses. According to the results of our surveillance, rMDPV was not detected in local waterfowl populations in Taiwan before this investigation. Therefore, it is unlikely that the 20-0910G was generated directly from local parvovirus strains in Taiwan. Wild geese are the likely hosts for infective GPVs [31]. Therefore, wild waterfowl could be vectors for long-distance spread waterfowl parvoviruses during seasonal migration. However, the route for 20-0910G transmitted to the goose farm in Taiwan requires further investigation.

GPVs induced clinical signs and mortality in both geese and Muscovy ducks, while MDPVs have caused disease so far only in Muscovy ducks. Geese can have asymptomatic MDPV infection, but shed the virus from the cloaca [5,6]. The rMDPVs, such as JH06 and JH10 strains, are highly pathogenic to Muscovy ducklings [19]. Our investigation showed that 20-0910G strain killed goose embryos and goslings within 11 and 12 days post-inoculation, respectively, indicating that rMDPVs were highly virulent to ducklings as well as to goslings (Figure 3).

Recombination is an important mechanism of virus evolution. New variant strains derived from recombination can broaden the host ranges and help the virus to escape from immune responses to cause outbreaks of disease in vaccinated hosts [32,33]. rMDPVs were generated from two recombination events and then these recombinant viruses spread and caused epidemics in different parts of the world. The 1.1-kb recombination region encoding the VP3 protein of 20-0910G was derived from a classical GPV strain. VP3 is a main structural protein of waterfowl parvoviruses that is critical for host range and pathogenicity. The presence of GPV-like VP3 could be a reason that the rMDPVs, like 20-0910G, can readily infect geese. Further investigation is required to address this issue.

## 5. Conclusions

In the current study, a recombinant waterfowl parvovirus, 20-0910G, was isolated in a goose flock in Taiwan. This rMDPV was derived from a recombination between the classical MDPV and the classical GPVs at the P9 promoter and partial VP3 region. Phylogenetic analysis revealed that 20-0910G clustered with the Chinese rMDPVs and its genomic sequence has a high degree of sequence identity to the JH10 strain. Animal experiments revealed that 20-0910G was highly pathogenic to goose embryos and goslings, with 100% mortality in challenged birds. Epidemiologic and serological analyses are necessary to elucidate the characteristics of infection in the fields and of cross-reaction to classical GPVs and MPDVs.
